# Experimental Manipulation of Dispersal Ability in A Neotropical Butterfly *Anartia fatima* (Lepidoptera: Nymphalidae)

**DOI:** 10.3390/insects9030107

**Published:** 2018-08-22

**Authors:** Robert B. Srygley

**Affiliations:** 1 Smithsonian Tropical Research Institute, Apdo. 2072, Balboa, Republic of Panama; 2 USDA-Agricultural Research Service, Northern Plains Agricultural Research Lab, 1500 N. Central Ave., Sidney Montana, MT 59270, USA; robert.srygley@ars.usda.gov; Tel.: +1-406-433-9420

**Keywords:** flight muscle ratio, flight performance, conservation, migration, mark release recapture

## Abstract

Research on endangered British butterflies has found that butterfly populations in small refuges evolve to allocate more mass to the thorax (flight muscle) and less to the abdomen than populations in large refuges. The observed change in mass allocation affects two morphological features relevant to flight: the flight muscle ratio (FMR) and the position of center of body mass (cm_body_). The author tested whether a decrease in FMR or a change in cm_body_ reduced the ability to disperse by experimentally weight-loading Neotropical *Anartia fatima* butterflies. In one treatment group, FMR was decreased but cm_body_ was not altered, whereas in the second group FMR was decreased and cm_body_ was repositioned further posterior. In one mark–release–recapture (MRR) experiment, butterflies dispersed relatively slowly, and treatment groups did not differ significantly. In a replicate experiment, butterflies dispersed more quickly, and control butterflies dispersed more rapidly than either treatment group. Differences in dispersal were consistent with a causal relationship between FMR and movement. A more posterior cm_body_ had little effect on dispersal beyond that due to the change in FMR. These results support the hypothesis that an increase in mass allocation to the thorax in small, dispersed refugia is due to selection on the ability to disperse.

## 1. Introduction

The ability of a species to disperse is a key feature that can define the species range [1], allow persistence in fragmented habitats [2,3], or allow the species to track changes in climate [4]. Among British butterfly species, regional extinction rates are greatest for species with intermediate dispersal ability, in part because their requirements for larger areas of suitable habitat make emigration from small local populations and regional extinction more likely than sedentary species [2]. For grasshoppers in Germany, low dispersal ability enhances the extinction risk of rare species [5].

For insects dispersing in the flight boundary layer [6], the ability to disperse is likely to be related to the insect’s flight speed and flight morphology [7]. Among butterfly species, an increase in flight speed is associated with an increase in mass allocated to the thorax [8,9]. An inverse relationship between mass allocated to the thorax (flight muscle) and abdomen (reproductive and digestive tissues) results in a potential trade-off between flight performance and reproduction [10,11]. Differences in morphology are also found both within and among species that differ in mating tactics. Butterflies that perch and then accelerate to intercept conspecifics have more mass allocated to the thorax than those which patrol [12,13] with mass allocation to the thorax directly proportional to the capacity to accelerate [14].

Both mass allocation to the thorax (i.e., the residuals of the linear regression of thoracic mass on body mass, [10]) and flight muscle ratio (FMR = thoracic mass/body mass) are measures of the body mass-specific power available for flight. FMR is related to linear acceleration and the ability to accelerate vertically against gravity to escape from predator attacks [11]. Mass allocation to the thorax is correlated with flight speed [9], suggesting that a larger thorax is associated with greater muscle cross-sectional area and hence, greater contractile force [10].

The position of center of body mass (cm_body_) is also associated with mass allocation to the thorax [9]. Theoretically, the position of cm_body_ relative to the wing base is directly related to the insect’s maneuverability [8,9,15]. This hypothesis is supported by the extremely high correlation among butterfly species between cm_body_ and the moment of rotational inertia (RI), a measure of the response of a butterfly’s body to the torque applied by the wings [16]. Theoretically, a lower RI permits faster radial acceleration and greater maneuverability for any given aerodynamic force. Therefore, these two traits, mass allocation to the thorax, or FMR, and cm_body_, both have theoretical and empirical relevance to flight performance. However, they are also highly correlated with one another [16].

Mass allocation to the thorax and abdomen changes in endangered species with restricted ranges and species with recent range expansion. For endangered *Plebejus argus*, mass allocation to the thorax increased and that allocated to the abdomen declined as the area of limestone habitat declined [17,18]. Mass allocation to the thorax declined with an increase in refuge habitat area for endangered *Hesperia comma* [19], but mass allocation to the thorax increased in areas recently colonized by *Pararge aegeria* [20]. Presumably local variation in the relative benefit of improved flight performance such as dispersal may select for an increase in mass allocation to the thorax (see also [21]). However, in these correlational experiments, both FMR and the position of cm_body_ change with mass allocation to the thorax.

In order to test the effects of FMR and the position of cm_body_ on dispersal, the author experimentally manipulated these traits by adding weights to the banded peacock *Anartia fatima* butterflies. Increasing weight-loading at any position has two potential effects on flight. The first is a decrease in the ability to accelerate forward resulting from a decline in FMR, and a decrease in forward flight speed resulting from a decrease in the mass-specific power available for flight. Repositioning cm_body_ further posterior has been predicted to result in a decrease in maneuverability [9,16,22,23]. Hence, repositioning cm_body_ should not affect dispersal more than the addition of weight at the position of cm_body_.

## 2. Materials and Methods

### 2.1. Study Organism and Study Site

*Anartia fatima* Godart (Nymphalidae, Lepidoptera) is found exclusively in heavily disturbed areas of lowland Neotropical rainforest where immatures feed on *Blechum* sp. (Acanthaceae) and adults feed on nectar. The species is not known to be seasonally migratory in Panama, although they have been observed crossing gaps greater than 1.5 km, such as Lake Gatún (personal observation). Airspeeds of *A. fatima*, undistracted from a generally straight flight path over Lake Gatún, averaged 3.95 m/s [9]. Further details on the life history and behavior of *A. fatima* may be found in [24].

**Figure 1:**
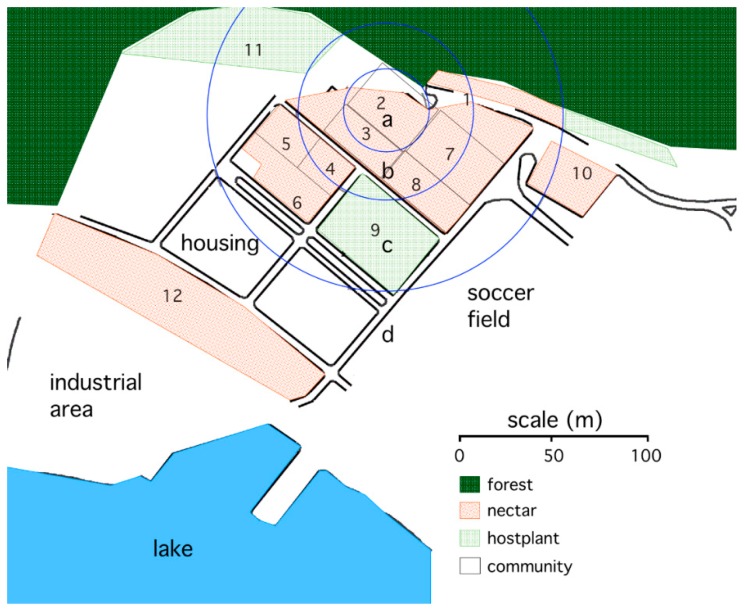
Map of the study site in Gamboa, Panama, and general characteristics of the habitats. Recapture sites (numbered areas) were classified into one of four zones (a through d) defined by concentric circles measured from the point of release to the center of the site. Habitats abundant with nectar and hostplants were bounded by habitats of poor quality for the butterflies, including forest, community housing, grass parks, and the lake.

The study site where butterflies were captured and released for mark–release–recapture studies was an abandoned settlement on the edge of Gamboa, Panama (see Figure 1). The study site was artificially divided into blocks by asphalt roadways. The blocks were overgrown with a mixture of old pasture and young secondary forest stands. An abundance of *A. fatima* butterflies inhabited this area, feeding on adult nectar plants (*Lantana horrida*, milkweeds, and composites) in the old pasture and laying eggs on their hostplant along the shaded pasture edge. The study site was bounded by areas where resources for the butterflies were rare: old secondary forest on a steep hillside to the north and east, and Lake Gatún (forming a part of the Panama Canal) and asphalted industry or community areas to the south and west. The roadways within the study area served to divide the area into 12 sectors that were subsequently simplified into four zones (see Figure 1 below).

### 2.2. Experimental Treatments

During the morning hours (08:00–12:00), adult butterflies were hand-netted on the study site, and handled such that damage and desiccation were minimized. Only those in reasonably young condition (e.g., freshly emerged or intermediate-fresh with little loss of wing scales) and occasionally those with intermediate wing-wear were used in the experiments. Butterflies were kept in humid Ziploc bags for up to 3 h in the field, and then placed in refrigeration or on ice in the nearby laboratory for up to 3 h until measurements and manipulations were performed. Each butterfly was sexed and weighed on a Sartorius balance (accuracy ±0.5 mg). It was then held by the wings on a mounting board using a pair of spring-loaded hairpins fixed to the board, and the body length (L_body_) was measured to the nearest 0.5 mm. 

In order to reposition cm_body_ further posterior by approximately two standard deviations (i.e., to an extreme of natural variation), 15% of the body mass of each individual (rounded to the nearest milligram) in the form of a pre-cut tin-alloy solder weight (Alpha Metals Inc., Providence, Rhode Island, USA: 0.050 American Wire Gauge, 60% tin 40% lead with rosin flux core) was glued using Duro contact cement to a position on the abdomen corresponding to 26% of L_body_ from the wing base. This position for the additional mass was 10% further posterior than the mean cm_body_ for *A. fatima* at 16% of L_body_ from the wing base. This experimental group was termed center of mass (CM). Weight-loaded butterflies (WL) were treated identically, except that the weight was added at cm_body_ (16% of L_body_ from the wing base). Unweighted controls were treated in the same manner, except that contact cement only was placed on the abdomen at 26% of L_body_.

In a related experiment, measurement of 52 *A. fatima* butterflies demonstrated that the position of cm_body_ for CM males shifted 2.0 s.d. posterior to that of the same males prior to manipulation and FMR decreased 1.5 s.d. The position of cm_body_ for WL males was 0.21 s.d. anterior to that for the same males prior to manipulation, and FMR decreased 1.5 s.d. (for details, see [23]).

### 2.3. Dispersal

In order to measure dispersal, two mark–release–recapture experiments (MRR) were performed in 1996: the September experiment occurred between 18 September and 1 October, and the October experiment, between 30 September and 8 October. Releases occurred during five of the first eight days of the first experiment (18–25 September 1996) and during the first three days of the second experiment (30 September–2 October 1996).

Following the manipulations, butterflies were uniquely numbered on the ventral surface of both hindwings using a silver paint marker. When the paint had dried, the butterflies were placed in glassine envelopes, re-weighed, returned to a nylon net bag, and transported to the field. The butterflies were released near a *Lantana* shrub at the center of zone a (see Figure 1) between 15:00 and 17:00 h. During the release, the butterflies’ proboscises were hand-extended into a 20% sucrose solution upon which they were permitted to feed and then fly away of their own volition. For the September experiment, 17 of 295 butterflies were not released (eight control, four WL, and five CM) because they were damaged. For the October experiment, two butterflies (both WL) out of 179 were not released.

In order to measure dispersal in the field, butterflies were resighted or recaptured (resampled). When resampled, each butterfly’s unique number was noted. The resampling site and the activity when first observed (flying, nectaring, basking with wings open, resting with wings closed, or courting/mating) were also recorded. If possible, treatment was also noted so that butterflies that had lost their weights could be recaptured and re-weighted. However, no butterflies with missing weights were observed in either experiment. Resampling generally occurred in the morning between 08:30 and 12:30. From one to four people resampled the population each day for a total of 121 person-hours during the period of 19–30 September 1996 and 68 person-hours during the period of 1–8 October 1996. Each person was designated a sector or sectors of the study site that they sampled for 1 h and then rotated with the other people in order to avoid overlap.

To test for differences in dispersal among the treatment groups, resamplings of an individual on the first six dates following its release were tabulated for each of the experiments. If an individual was resampled more than once on a particular date, then one resampling site for that date was selected at random. Sites were classified into four zones (a–d), based on the distance from the point of release to the middle of the site (see the map, Figure 1). Zone a included sites that were within 70 m of the release point (sites 2 and 3); zone b included sites from 70 to 100 m distant (sites 1, 4, 5, 7, and 8); zone c included sites from 100 to 250 m distant (sites 6, 9, and 11), and zone d included sites from 250 to 350 m distant (sites 10 and 12). Analysis of deviance was used to test whether counts varied with sex, treatment, distance, and days following release; distance and days following release were entered as ordered categorical variables. Analysis of deviance, where deviance is defined as two times the log-likelihood, is the count data analogue for analysis of variance [25]. A significant interaction between treatment and distance or a three-way interaction among treatment, distance, and days following release indicated that different treatments dispersed differently.

In order to estimate dispersal, the author assumed that the passive diffusion model was applicable to the first three days following release, and estimated the dispersal constant D = d^2^/4t, where d^2^ is the mean squared displacement of released individuals at time t [26,27]. The study site was similar in shape to a semicircle bounded by less suitable habitat, whereas the model assumed that it was infinitely planar in form. Hence, dispersal distances were not normally distributed, and the results for dispersal distance should be interpreted with caution.

## 3. Results

During September, butterflies in different treatment groups did not disperse at significantly different rates, made evident by a lack of significant interaction between treatment and distance (see Table 1). The dispersal constant D was not significantly different among treatments, and the mean D for all individuals was 728 m^2^/day (s.d. = 94). 

**Figure 2:**
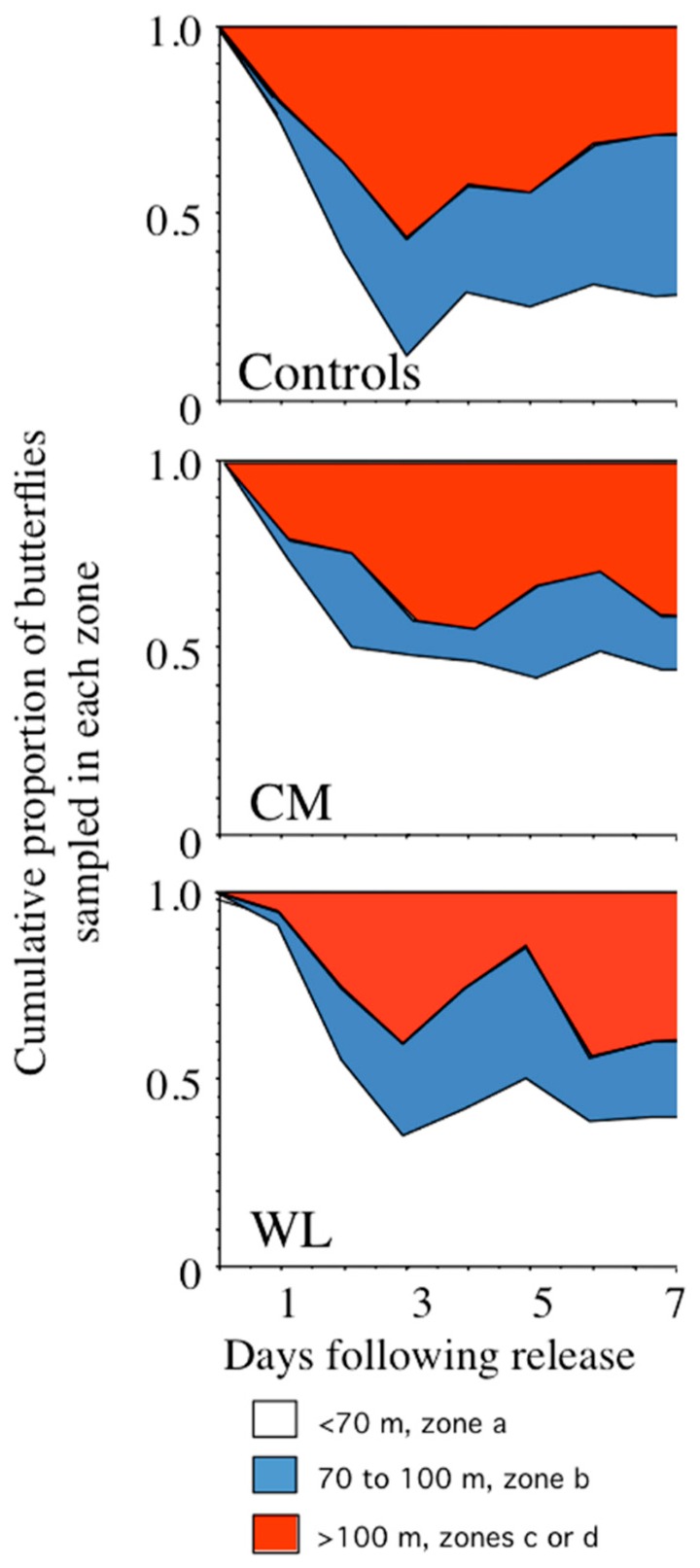
The cumulative proportion of resampled butterflies in zone a, which was nearest to the release point, to zones c and d, which were furthest from the release point, on days 1–7 following the releases in October. The zones refer to concentric circles measured from the release point in the middle of zone a (see Figure 1). Dispersal from the center is evident from a diminishing proportion of butterflies in zone a, and increasing proportions of butterflies in zone b or zones c and d. Control butterflies dispersed faster than either center of mass (CM) or weight-loaded (WL) butterflies, particularly during the first three days following release. WL, group of butterflies for which weights were added near to cm_body_; CM, group of butterflies for which weights were added posterior to cm_body_.

During October, butterflies in different treatment groups dispersed at significantly different rates (see Figure 2) evident by a significant interaction between treatment and distance (see Table 2). This difference in dispersal was primarily due to control butterflies moving further than either WL or CM butterflies during the first three days following release. The dispersal constant D was highly variable both within and among treatment groups. On average, D was greater for control butterflies (mean D ± s.d., 1529 ± 25 m^2^/day) than for CM (1117 ± 25 m^2^/day) and WL butterflies (1329 ± 1082 m^2^/day). This reduction in D of both CM and WL butterflies relative to controls serves as a confirmation of the difference in dispersal rates found in the qualitative analysis (see Figure 2).

**Figure 3:**
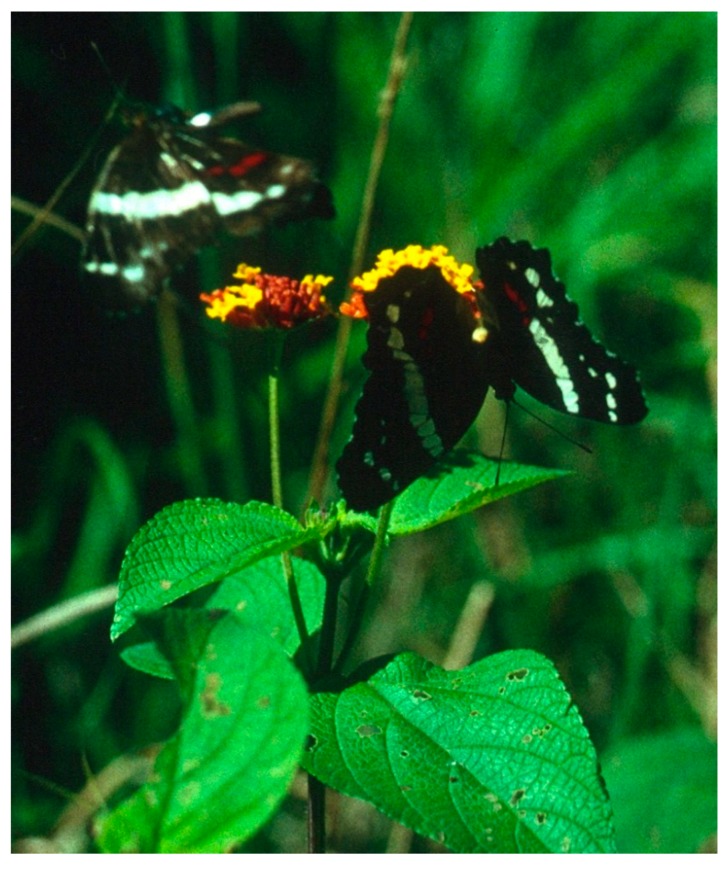
A control *A. fatima* male courts a CM female on a *Lantana* flower.

In general, dispersal distances were much lower in September than October (see Table 3 and Table 4), which may have been due to overcast conditions during the former month. The weather during September also differed from that during October. It was overcast on three of the 13 sampling dates in September, and mostly cloudy (covering greater than 50% of the visible sky) on another three dates. In October, it was mostly cloudy on only two of the eight sampling dates and never overcast. In both months, the behaviors of *Anartia* were significantly dependent on the weather (log-likelihood tests: *p* < 0.0001). Behaviors also differed significantly between experiments (log-likelihood test: *p* < 0.004). An increase in the proportion of time that butterflies were courting/mating (see Figure 3) in the second experiment contributed the most to this difference.

The study site was surrounded by less favorable habitats for the butterflies, and this may have served to minimize dispersal out of the study site (see Figure 1). As evidence that butterflies did not disperse regularly out of the study site, few marked butterflies were captured in the flower-rich habitat near the lake shore (site 12 in zone d), even though a large fraction of the butterflies used in these experiments were captured in this highly suitable habitat.

## 4. Discussion

The author predicted that altering the position of cm_body_ would not affect flight speed and thereby the dispersal of butterflies, whereas the addition of weight would decrease dispersal. In the October experiment, when the dispersal constant was greater than 1000 m^2^/day, the experimental addition of weights resulted in a reduction in dispersal relative to controls. This experimental result indicated a causal relationship between mass allocation to the thorax and dispersal rate. Although cm_body_ moves posterior with increased allocation of mass to the abdomen, experimentally changing cm_body_ had little qualitative effect on dispersal. In a related paper, flight speed of released *A. fatima* did not differ between treatment groups [23]. Therefore, mass allocation to the thorax may be affecting dispersal rate independent of flight speed.

Treatment effects were not significantly different in September during relatively poor environmental conditions for flight. Compared to October, September weather conditions were more frequently overcast and dispersal rates were lower. It is likely that the lower dispersal rates for September were due to weather conditions unsuitable for flight. *Anartia fatima* are palatable and less prone to fly when direct sunlight is not available [28]. For example, *Anartia* was more likely to be observed resting or basking in overcast conditions in September when conditions were poor for flight. In addition, the frequency of flying and nectaring increased with the amount of sun. Hence, butterflies may not have dispersed as far in September because nectar was available near the release site. Perhaps as a result, control butterflies dispersed at the same slow rate as weight-loaded ones. However, compared to weighted (WL + CM) butterflies, control butterflies that were known to be alive in September were less likely to be resampled [23], which suggests they either fled or hid when approached.

In October when it was never overcast, *Anartia* were more likely to be observed basking and never observed flying in full sun when less than 10% of the sky was covered by clouds. Nectaring was most frequently observed in mostly sunny conditions when clouds covered less than 50% of the visible sky. With poorer conditions, the frequency of flying or resting tended to increase again. In October, when weather conditions were more prone to activity, control butterflies appeared to have dispersed further to nectar, search for hostplants, court, and mate. There was no difference among treatment groups in the probability of resampling butterflies that were known to be alive in October [23].

In comparison to other butterflies, *A. fatima* butterflies disperse slowly. Dispersal constants ranged from 700–2500 m^2^/day. Dispersal rates from different studies are difficult to compare because time and dispersal distance are uncorrelated, causing variation to be heavily dependent on the duration of the study. In addition, the distance moved per unit time may vary with location in a heterogeneous landscape, resulting in within- and between-habitat rates of movement (C. D. Thomas, personal communication). The behavior of butterflies at the edge of a suitable habitat, for example, whether they cross large gaps of an unsuitable habitat, settle on the boundary, or reflect back toward the center of the suitable habitat, may also differ among species (see [29] for responses to habitat heterogeneity). With those caveats in mind, dispersal for a temperate pierid butterfly *Aporia crataegi* was between 10,000–86,000 m^2^/day [30]. For experimentally released lycaenid butterflies *Plebejus argus*, median dispersal distances within a patch were 2465 m^2^/generation for males and 200 m^2^/generation for females (mean adult longevity is four days, [31]). The low dispersal rate of *A. fatima* should make it particularly amenable to further experimental studies.

Because of the trade-off between mass allocation to the thorax and the abdomen, the ability to disperse may have a reproductive cost. A similar trade-off has been attributed to the additional somatic maintenance associated with a prolonged lifespan [32] in other populations. Hibernating comma butterflies *Polygonia c-album* allocate more nitrogen to thoracic tissue and less to abdominal tissue than direct-developing butterflies [33]. Although there is no direct evidence that hibernating *P. c-album* are more dispersive, it is likely that allocation of mass is associated with dispersal in the population. The butterflies are bi-voltine. Early summer immatures allocate more to reproduction, because when they emerge as adults, they have the same hostplants (Urticaceae) upon which they fed as larvae available in their immediate vicinity. Late summer individuals allocate more to thoracic muscle, because when they emerge from hibernation as adults, local environmental conditions may no longer be favorable for hostplants and dispersal may be warranted.

## 5. Conclusions

These results from experimentally weight-loaded *A. fatima* support the hypothesis that an increase in mass allocation to the thorax in small, dispersed refugia [17,18,19] and in recently expanded ranges [20] was due to selection on the ability to disperse. Because dispersing butterflies allocate less mass to the abdomen, species that are expanding their range to track climate change or persisting in fragmented habitats via migration between suitable patches are also the ones that might have less reproductive capacity. This reproductive cost may increase the risk of extinction for populations with restricted ranges that migrate among less persistent habitats (e.g., endangered *Plebejus argus* and *Hesperia comma* [17,18,19]) or founder populations expanding the species’ range to track changes in climate. Some butterfly species are able to mitigate this reproductive cost by reallocating mass from the thorax to reproduction as they age [34].

In comparison to many other tropical butterfly species, *A. fatima* butterflies are common in disturbed habitats. Their short lifespan, limited dispersal distances, ease of obtaining large sample sizes in a brief amount of time, and the high probability of resampling (45–85%, [23]) without direct interference (e.g., resightings rather than recaptures) make *Anartia* butterflies particularly amenable to experimental analyses of dispersal.

## Figures and Tables

**Table 1:** Analysis of deviance of *Anartia fatima* dispersal data from September.

Factor	d.f.	Deviance	d.f.	Residual Deviance	*p* ^1^
Null model			131	471.0	
Sex	1	80.4 ^1^	130	390.6	0.0000
Distance	3	173.7	127	216.9	0.0000
Capture date	6	59.1	121	157.8	0.0000
Treatment	2	3.1	119	154.7	0.215
Sex × Distance	3	2.7	116	152.1	0.446
Sex × Capture date	6	4.7	110	147.4	0.581
Distance × Capture date	18	83.0	92	64.4	0.0000
Treatment × Distance	6	7.4	86	56.9	0.281
Treatment × Capture date	12	4.4	74	52.5	0.995
Sex × Distance × Capture date	17	8.0	57	44.5	0.965
Treatment × Distance × Capture date	30	24.4	27	20.1	0.754

^1^*p*-values are results from a X^2^ test for each factor entered sequentially.

**Table 2:** Analysis of deviance of *Anartia fatima* dispersal data from October.

Factor	d.f.	Deviance	d.f.	Residual Deviance	*p* ^1^
Null model			131	217.7	
Sex	1	45.5	130	172.2	0.0000
Distance	3	58.6	127	113.6	0.0000
Capture date	6	21.3	121	92.3	0.0016
Treatment	2	0.5	119	91.8	0.783
Sex × Distance	3	1.8	116	89.9	0.604
Sex × Capture date	6	6.6	110	83.4	0.363
Distance × Capture date	17	33.4	93	50.0	0.010
Treatment × Distance	6	13.4	87	36.6	0.037
Treatment × Capture date	12	3.0	75	33.6	0.995
Sex × Distance × Capture date	16	4.4	59	29.2	0.998
Treatment × Distance × Capture date	30	16.5	29	12.6	0.978

^1^*p*-values are results from a X^2^ test for each factor entered sequentially.

**Table 3:** Mean squared distances (d^2^) and dispersal constants (D) of *Anartia fatima* during the first three days following their September release.

Day	Treatment	d^2^	s.d.	D (m^2^/day)
1	Control + CM + WL ^1^	2813	8251	703
2	Control + CM + WL	5199	9950	650
3	Control + CM + WL	9993	18,209	832

^1^ Treatments were pooled because no significant difference was found among them.

**Table 4:** Mean squared distances (d^2^) and dispersal constants (D) of *Anartia fatima* during the first three days following their October release.

Day	Treatment	d^2^	s.d.	D (m^2^/day)
1	Control	4212	7151	1053
2	Control	10,802	9169	1350
3	Control	26,193	25,561	2183
1	CM	4394	7160	1098
2	CM	9169	18,167	1146
3	CM	13,294	20,883	1108
1	WL	1160	3140	290
2	WL	19,593	33,939	2449
3	WL	14,968	20,734	1247

## Supplementary Materials

Supplementary File 1
